# Influence of formulation on mobility of metazachlor in soil

**DOI:** 10.1007/s10661-014-3633-9

**Published:** 2014-02-06

**Authors:** Małgorzata Włodarczyk

**Affiliations:** Faculty of Environmental Management and Agriculture, West Pomeranian University of Technology in Szczecin, ul. Słowackiego 17, 71-176 Szczecin, Poland

**Keywords:** Metazachlor, Formulation, Mobility, Soil, Sodium alginate, Column

## Abstract

The mobility of metazachlor [2-chloro-*N*-(pyrazol-1-ylmethyl)acet-2′,6′-xylidide] in sand soil and loamy sand soil was studied in a soil column under laboratory conditions. Commercial metazachlor formulation (Metazachlor 500 suspension concentrate (SC)) and metazachlor immobilized in alginate matrix were used for leaching experiment. The initial concentration of metazachlor in soil for both formulations was 2.0 mg mL^−1^. After application of herbicide, the columns were irrigated with 100, 40, and 3.7 mm of water. After 1 h, when addition of water was completed, soils were sampled in 5-cm segments and were used for the analysis of residues. The use of alginate controlled release (CR) formulation reduced the vertical mobility of metazachlor into soil layer in comparison with the formulation SC.

## Introduction

The application of chemical crop protection contributes to the intensification of food production; however, it is a source of anthropogenic pollution. A strong influence on behavior of an active substance in the environment is the formulation of a pesticide, whose components such as synergists, buffers, activators, organic solvents, adsorbents, fillers, or adjuvants, modify the physical and chemical properties of the active substance (Mulqueen 2003; Green and Beestman 2007; Knowles 2008). According to numerous studies, these compounds have an effect on availability, durability, mobility, and, in consequence, biologic characteristics of the pesticides (Reddy 1993; Beulke and Malkomes 2001; El-Nahhal 2003; Sondhia 2009; Chopra et al. 2010; Kucharski and Sadowski 2011). Furthermore, the application rates for conventional pesticide formulations, during the time required for weed control, are generally higher than the minimum threshold to counter losses from degradation, leaching, volatilization, and adsorption (Maqueda et al. 2009). It was found, that less than 10 % of the pesticide applied actually reaches the target pest, and the rest penetrates the environment unnecessarily, contaminating the soil, water, and air, thus affecting some nontarget organisms (Mogul et al. 1996; Arias-Estevez et al. 2008).

The use of controlled release (CR) technology could solve the problem of low efficacy and environmental pollution resulting from the use of conventional pesticide formulation. CR formulation can reduce the surface runoff and leaching of soil by applied pesticides and decrease the amount of pesticide being applied to the soil. Additionally, the use of controlled release formulation may reduce the losses to evaporation and photolysis (Mogul et al. 1996; El-Nahhal et al. 1999; Fernandez-Perez et al. 2000, 2011; Flores-Cespedes et al. 2007).

The parameters that effects the properties of CR formulation depend on the nature and type of polymer used. The natural polymers such as alginate, ethylcellulose, starch, lignin, and chitosan are preferred to use in CR formulations in agriculture, because of their nontoxic, low cost, availability, and biodegradability characteristics (Flores-Cespedes et al. 2007).

Alginate gel has been used as a matrix for preparing controlled release formulation of herbicides. Additionally, use in researches on modifying agents such as natural and activated clays, activated carbon, humic acid, or linseed oils in the preparation of CR formulations from alginate caused an increase in the efficiency of encapsulation process and better control on the release profile of active ingredients. Pepperman and Kuan (1993, 1995) found that the use of linseed oil and alginate as the basis of a CR formulation of alachlor and metribuzin results in reducing the release of the herbicide in comparison with conventional formulation. The leaching potential of atrazine alginate linseed oil CR formulation was investigated by Johnson and Pepperman (1995). It was found that atrazine CR formulations with and without linseed oil leached significantly less than a liquid atrazine formulation based on a technical material. Laboratory tests showed that modifying agents like bentonite, anthracite, and active carbon of the alginate formulations reduce the release rate of the chloridazon, metribuzin (Fernandez-Perez et al. 2000), or isoproturon (Flores-Cespedes et al. 2007) in comparison with the technical products and with alginate formulations without modifying agents.

The herbicide metazachlor [2-chloro-*N*-(pyrazol-1-ylmethyl)acet-2′,6′-xylidide] is a commonly used preemergent herbicide used to inhibit growth of plants especially in rape culture. It is a relatively nonpersistent compound (DT_50_ in soil from 3 to 9 days), but occurs in surface and ground water due to spray-drift or runoff in concentrations up to100 μg L^−1^ (Mohr et al. 2007).

The aim of the present study was to determine the influence of formulation on mobility of metazachlor in the soil.

## Materials and methods

### Chemicals

Two formulations of herbicide metazachlor were used in the experiment: suspension concentrate (SC), as a commercial preparation of Metazachlor 500 SC, and in the form of capsules based on the alginate matrix. The alginate capsules of metazachlor were obtained in the Center of Bioimmobilization and Innovative Packaging Materials at the West Pomeranian University of Technology in Szczecin, Poland (Włodarczyk et al. 2009). Calculated to 1 g of the capsule, metazachlor content in the alginate capsule was 52.77 mg s.a.

The active substance, with purity of 98.6 %, was provided by Feinchemie Schwebda GmbH, Germany. Selected physical and chemical properties of metazachlor are presented in Table 1.

**Table 1:** Selected physical and chemical characteristics of metazachlor (Mohr et al. 2007; http://www.fao.org/ag/AGP/agap/Pesticid/Specs/docs/Pdf/new/metazach.pdf)

Structure diagram	IUPAC name	2-chloro-*N*-(pyrazol-1-ylmethyl)acet-2′,6′-xylidide
	Physical state	Colorless crystals
	Chemical formula	C_14_H_16_ClN_3_O
	Molecular mass	277.8 g mol^−1^
	Solubility in water	450 mg L^−1^ (20 °C)
	Octanol/water partition coefficient	log *K*_ow_ = 2.49

### Soils

Two soils were used in this experiment. Both soils were taken from Western Pomerania Region, Poland. Soil samples were chosen according to their content of organic carbon and collected at a 0–20-cm depth, air dried, homogenized, and passed through a 2.0-mm sieve. The physicochemical and granulometrical characteristics of these soils are given in Table 2.

**Table 2:** Selected properties of soils

Soil	Granulometric group	Water capacity	Organic carbon	Nitrogen	Sulfur	pH	pH
		[%]	[%]	[%]	[%]	H_2_O	KCl
G1	Loamy sand soil	34.63	0.83	0.075	0.0076	5.56	4.28
G2	Sand soil	37.18	1.99	0.159	0.0232	4.56	3.71
Soil	Hh	CEC		TEB		BS	
	cmol/kg	cmol/kg		cmol/kg		[%]	
G1	3.33	16.10		19.43		82.88	
G2	10.33	15.30		25.63		59.71	

*Hh* hydrolytic acidity—the hydrogen and aluminum ions, exchangeable and nonexchangeable, bounded by the soil sorption complex, and is the sum of all of the hydrogen ions in the soil; *CEC* cation exchange capacity—the maximum quantity of total cations that a soil is capable of holding at a given pH value, available for the exchange with the soil solution; *TEB* total exchangeable bases—the sum of exchangeable cations (Ca^2+^, Mg^2+^, K^+^, Na^+^) excluding Al^3+^ and H^+^; *BS* base saturation—the ratio of the quantity of exchangeable bases to the cation exchange capacity (Osman 2013)

### Mobility experiment

Mobility of metazachlor in soil was tested using PCV columns (ca. 40 cm long, diameter of *Φ* = 3 cm) filled with 150 g of soil. The height of the soil stack in the column was 20 cm. Both herbicide treatments (solution of Metazachlor 500 SC and alginate capsules) were applied to triplicate soil columns. For both formulations, the same concentration of the active substance was applied *C*_0_ = 2.0 mg mL^−1^. Then, the columns were irrigated with a dose of water corresponding to the amount of rainfall characteristic for Western Pomeranian Region, Poland: 100 mm (maximum rainfall), 40 mm (average maximum rainfall), and 3.7 mm (average rainfall). Having completed the application of water, after 1 h (to minimize the degradation process), the content of the column was divided into 5-cm pieces, for which the active substance concentration was determined (0–5 cm for layer I, 5–10 cm for layer II, 10–15 cm for layer III, and 15–20 cm for layer IV). In case of the maximum rainfall, water elutes (layer V) collected during the experiment were also subjected to extraction.

### Chemical analysis

The wet soil samples were extracted with acetone in a mechanical shaker for 4 h and filtered. Then, acetone was evaporated in a rotary vacuum evaporator (35 °C), and residues (water samples) were liquid-liquid extracted with chloroform. Extracts were dehydrated with anhydrous sodium sulfate, purified on columns filled with florisil and anhydrous Na_2_SO_4_, and evaporated to a minimum volume (2 mL) for analysis. Water elutes (fraction V) were liquid-liquid extracted with chloroform, dehydrated with anhydrous sodium sulfate, and evaporated to a minimum volume (2 mL) for analysis. Recovery of the active substance was as follows: for the alginate capsules, 94.99 ± 2.59 %, and for the SC formulation, 97.20 ± 2.39 %. All the measurements were recorded three times (Ambrus et al. 1981; Kucharski et al. 2010).

The recovery of metazachlor was determined by the fortification of soil samples at the concentrations of 0.01, 0.1, and 1.0 mg kg^−1^ and water samples at the concentrations of 0.01, 0.1, and 1.0 mg mL^−1^ in three replicates. The average recovery of metazachlor for the soil samples was 94 % and for water samples, 98 %. The quantification limit of the method for the soil samples was 0.0005 mg kg^−1^ and for water samples was 0.005 μg mL^−1^.

Gas chromatography was used to determinate concentrations of metazachlor. A PerkinElmer Clarus 600 gas chromatograph was equipped with an MS detector and an Elite 5MS column (30 m × 0.25 mm, 0.5 μm in film thickness). The operating temperatures were the following: detector of 320 °C, oven temperature of 100 °C for 1 min, 10 °C min^−1^ to 250 °C for 5 min, and 25 °C min^−1^ to 300 °C for 2 min. The carrier gas was helium with a flow rate of 1.0 mL min^−1^. Under these conditions, the retention time was 16.07 min. To determine metazachlor in the samples, the electronic ionization method, type El+, was used. Metazachlor qualitative analysis was based on the mass spectrum and ions which are characteristic for this compound: 81, 133, 209, and 277. Quantitative analysis was performed by a comparative method, based on the calibration curve (*y* = 358809∙*x* − 282.04; *n* = 7, *R* ^2^ = 0.9995).

### Statistical analysis

All experimental data were calculated using the statistical program Statistica 10.0 for Windows. Statistical analyses were performed using two-way analysis of variance (ANOVA) to determinate the formulation and the soil type effect on mobility of metazachlor in soil. Means were compared by Tukey’s test and expressed as mean ± standard deviation. Differences were considered to be significant at a significant level *p* = 0.05.

## Results and discussion

On the basis of the performed experiment, it was found that mobility of metazachlor in soil is determined by the pesticide formulation, dose of water, and the physical and chemical characteristics of the soil.

**Fig. 1:**
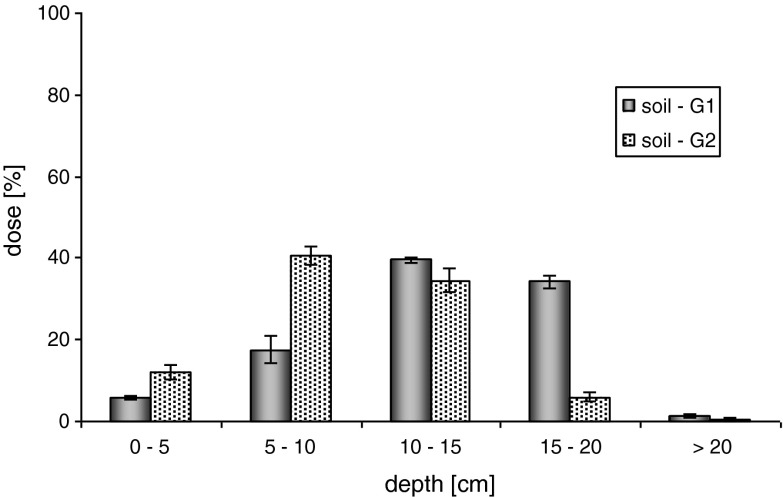
Mobility of metazachlor as formulation SC in soil—maximum rainfall (100 mm)

Regardless of the type of soil used in the experiment, a clearly higher mobility of metazachlor was observed in case of the SC formulation. As a result of application of a dose of water equal to the maximum rainfall (100 mm), the active substance was distributed among all the analyzed layers of the soil (Fig. 1).

For the loamy sand (G1), the highest metazachlor concentration was found in layers III (39.52 ± 0.74 %) and IV (34.22 ± 1.46 %), at the depth of 10–20 cm. In case of sand soil (G2), which has a higher content of organic carbon and a higher capacity of the sorption complex, the mobility of metazachlor in the soil proceeded at the slower rate. For soil G2, the highest metazachlor content was recorded for layers II (40.6 ± 2.23 %) and III (34.49 ± 2.91 %), at the depth of 5–15 cm. In the case of the maximum rainfall, the concentration of the active substance was determined for both soils in layer V (water elutes > 20) and amounted to G1 = 1.48 ± 0.52 % and G2 0.43 ± 0.45 %, respectively.

**Fig. 2:**
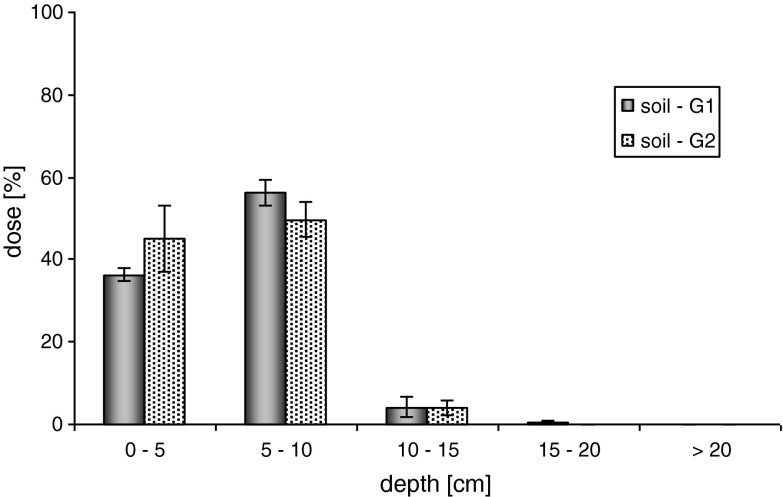
Mobility of metazachlor as formulation SC in soil—mean maximum rainfall (40 mm)

The application of the amount of water equals to the average maximum rainfall (40 mm) and retards the mobility of the herbicide confectionized in the SC formulation in the soil (Fig. 2). The highest concentrations of the active substance were determined in the first two soil layers, at the depth of 0–10 cm. A higher ability of metazachlor to move was plainly seen in the case of loamy sand soil G1, in comparison to sand soil G2. Its significant concentrations were measured in the range of 0–20 cm, while the highest recorded metazachlor concentration (56 % of the applied dose) recorded in the depth of 5–10 cm is much different from the concentration determined for layer I (according to the Tukey test, at *p* = 0.05). In case of soil G2, for layers I and II, two not significantly different metazachlor levels were measured, being 45.09 ± 7.85 % (1) and 49.75 ± 4.25 % (2), respectively. Additionally, no substantial concentration of metazachlor in layer IV, at the depth of 15–20 cm, was found.

The quantity of water equal to the average rainfall (3.7 mm) did not have any significant effect on mobility of metazachlor used in the form of commercial preparation Metazachlor 500 SC in the soil. After 1 h, the applied dose of water did not relocate itself below the first analyzed soil layer inside the column (0–5 cm).

**Fig. 3:**
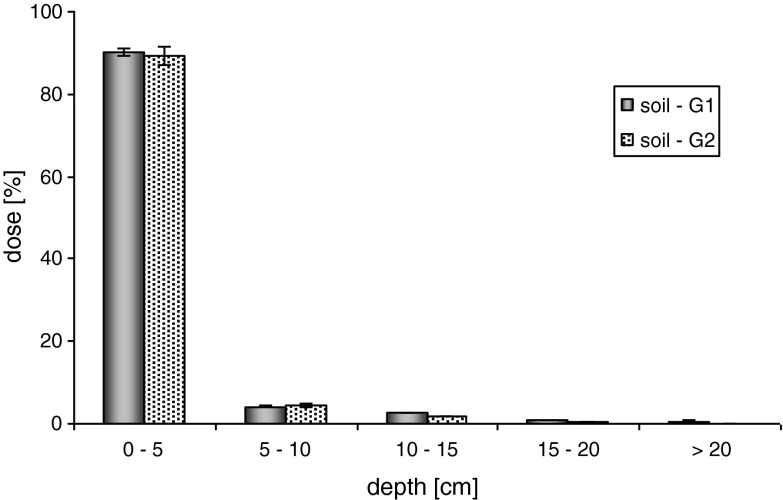
Mobility of metazachlor immobilized in alginate matrix in soil—maximum rainfall (100 mm)

**Fig. 4:**
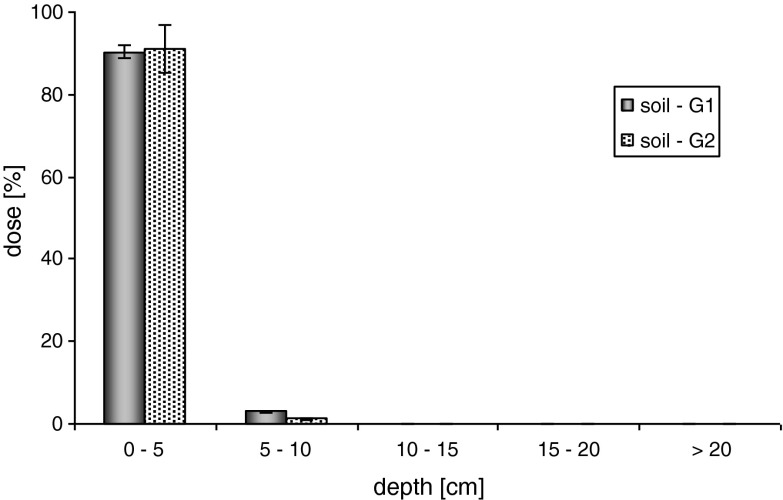
Mobility of metazachlor immobilized in alginate matrix in soil—mean maximum rainfall (40 mm)

Application of the alginate capsules in the experiment strongly reduced the mobility of metazachlor in the soil. Regardless of the water doses, the highest amounts of metazachlor, from 89.15 to 100.00 % of the applied initial concentration, were observed in layer I, at the depth from 0 to 5 cm. In the case of the maximum dose of water, metazachlor released from the alginate matrix (G1 = 8.33 ± 0.32 % and G2 = 6.48 ± 0.96 %) underwent distribution among the analyzed soil layers. For soils G1 and G2, its significant concentration was recorded for the depth of 5–10 cm (II)—around 4.0 % and for the depth of 10–15 cm (III)—approximately 2.0 %. In layers IV (15–20 cm) and V (eluate > 20 cm), the measured concentrations of the studied active substance remained below 1 % (Fig. 3). For the maximum average precipitation, metazachlor released from the alginate matrix (G1 = 3.01 ± 0.70 % and G2 = 1.55 ± 0.71 %) migrated to the depth of 5–10 cm. Below layer II, its significant content >1 % was not found. As for the SC formulation, the average precipitation did not have any stronger effect on metazachlor mobility, because the applied water dose did not penetrate below the first (0–5 cm) soil layer in question (Fig. 4).

As it was established during the experiment, the differences in the mobility of metazachlor applied in the form of Metazachlor 500 SC and the alginate capsules are significant and were confirmed in the Tukey test at *p* = 0.05.

The issue of herbicide leaching through soil is of great importance to a number of environmental and agronomical problems, just to mention groundwater contamination by herbicides. Such process is determined to a large extent by the physiochemical properties of herbicides and soils (mechanical composition, organic matter, soil moisture, pH), temperature and rainfall.

The metazachlor GUS index (leaching potential), calculated on the basis of literature data for half time (TD_50_ in soil 3–9 days) and distribution coefficient *K*_OC_ (54–80 mL g^−1^), indicates that metazachlor belongs to substances of low/intermediate leachability (GUS = 1.0–2.16) (Alister and Kogan 2006; Fernandez-Perez et al. 2011). Nevertheless, relatively large amounts of metazachlor are determined both in surface and ground waters (Mohr et al. 2007).

Based on own studies, it can be found that the formulation has a significant influence on behavior of metazachlor in the soil. Metazachlor marketed in the SC formulation features high mobility. This can be attributed to its high solubility in water (450 mg L^−1^) and relatively low value of coefficient *K*_OC_ < 100 mL g^−1^. That was confirmed in the studies carried out by Kucharski and Sadowski (2011) on the influence of adjuvants (Atpolan Bio 80 EC, Break Thru 240 EC, RackRow) on the behavior of metazachlor in the soil in the field conditions. Kucharski and Sadowski stated that adjuvants reduce the mobility of metazachlor in the soil, where metazachlor’s residues (14 weeks after the application) were determined at the depth of 31–50 cm only for those objects, where only metazachlor in the form of the preparation Butisan 400 EC had been applied. This was confirmed by studies performed during harvest when the residuals of herbicide were measured in the deeper soil strata (up to 0.0012 mg kg^−1^) in the objects free from any adjuvants. At the same time, the authors recorded some significantly longer time TD_50_ for metazachlor objects with the adjuvants.

The second formulation used in the experiment was based on the alginate matrix, and it significantly reduces the migration of metazachlor throughout the soil profile. This process depends on the applied dose of water (in the natural conditions—rainfall), or rather the amount of the active substance released from the matrix, of which behavior depends further on the physical and chemical properties of the soil. This notion is confirmed by, inter alia, studies conducted by Fernandez-Perez et al. (2000) on controlled release of imidacloprid from a lignin matrix and of diuron and isoproturon from a CR formulation, both obtained on the basis of alginate and bentonite and on their mobility in the soil environment. The authors indicate that the kinetics of releasing an active substance from a CRF matrix in the soil is several times slower than in water. This fact is attributed to the occlusion of the CRF matrix surface effectuated by the soil particles and the slower diffusion of the active substance from the matrix into the soil. Furthermore, the substances included in the soil solution may retard the migration of pesticides into the aquatic phase.

The same relationship was obtained by studying on the impact of the formulation on the mobility of pendimethalin in the soil (Włodarczyk 2011). Research shows that pendimethalin, as a commercial EC formulation (Panida 330 EC), has the ability to move in soil, which is confirmed by studies of Chopra et al. (2010). Use of pendimethalin immobilized in alginate matrix minimizes this process according to studies conducted (Włodarczyk 2011). Other researchers such as Fernandez-Perez et al. (2000, 2011), Flores-Cespedes et al. (2007), and Sopeña et al. (2007) also indicate that the use of formulations based on sorbents and alginate reduces the leaching of pesticide in the soil columns.

According to own research and the literature review, a continuous and intensive rainfall may result in translocation of pesticides into some deeper layers of the soil, which is of high significant importance due to the possible contamination of ground water. At the same time, application of formulations with a controllable release of the active substance may help us minimize this process considerably.
